# Author Correction: Fluctuating climate and dietary innovation drove ratcheted evolution of proboscidean dental traits

**DOI:** 10.1038/s41559-023-02204-8

**Published:** 2023-09-08

**Authors:** Juha Saarinen, Adrian M. Lister

**Affiliations:** 1 Department of Geosciences and Geography, University of Helsinki, Helsinki, Finland; 2 Natural History Museum, London, UK

**Keywords:** Palaeontology, Palaeoecology

Correction to: *Nature Ecology & Evolution* 10.1038/s41559-023-02151-4, published online 14 August 2023.

In the version of the article originally published, in the legend for Fig. 3i, “*P. macinnesi* (Amebelodontidae)” previously read “*P. macinnesi* (Gomphotheriidae)”. The sentence “Locality mean ordinated hypsodonty values were obtained from the NOW-database (data (https://nowdatabase.org/now/database/) by the NOW Community / CC BY 4.0), and from additional literature sources (see Supplementary Data 2)” has been moved from the fourth paragraph of “The ratchet effect of stepwise evolution under varying climate” to the second paragraph of “Stratigraphy and palaeonenvironments”. In addition, in the legend for Extended Data Fig. 9, “M3 (dext.)” has been corrected from “M3 (sin.)”. These changes have been made to the HTML and PDF versions of the article.

