# Supplementary material for: Fluctuating climate and dietary innovation drove ratcheted evolution of proboscidean dental traits
Source: Nat Ecol Evol. 2023 Aug 14;7(9):1490–502. doi: 10.1038/s41559-023-02151-4 (PMC10482678; doi:10.1038/s41559-023-02151-4)
Supplement: Supplementary file 1 — Supplementary Information Sections 1–6 (including Supplementary Analysis Tables 1–6) and references. [file 41559_2023_2151_MOESM1_ESM.pdf]

nature ecology & evolution

Article

https://doi.org/10.1038/s41559-023-02151-4

# Fluctuating climate and dietary innovation drove ratcheted evolution of proboscidean dental traits

In the format provided by the
authors and unedited

# This PDF includes:

Supplementary Information 1: Taxonomy of East African Proboscidea……………………………………………………………2

Supplementary Information 2: Molar morphology…………………………………………………………………………………..2

Supplementary Information 3: Supplementary Analyses……………………………………………………………………….…3

Supplementary Information 4: Regional changes in vegetation and proboscidean diets in East Africa during the Neogene……………………………………………………………………………………………………………………………………….11

Supplementary Information 5: Testing for correlated evolution of proal chewing and major steps in the dental trait evolution of Proboscidea………………………………………………………………………………………….……….12

Supplementary Information 6: Published data on East African Neogene palaeoenvironments……………..……15

References (81 – 177)…………………………………………………………………………………………………………………………..29

## 1. Taxonomy of East African Proboscidea

In the African Neogene, Proboscidea comprised the Deinotheriidae, and the elephantoid families Mammutidae, Stegodontidae, Gomphotheriidae, Choerolophodontidae, Amebelodontidae, Tetralophodontidae, Anancidae and Elephantidae (Extended Data Fig. 1). Many of these, apart from deinotheres, mammutids and elephants have been classed as "gomphotheres", but this is a paraphyletic assemblage of diverse buno-lophodont elephantoid proboscideans (*15, 81*), here treated as families. The stem-group of true elephants (Elephantidae), formerly considered the Stegodontidae (e.g. *82*), is now placed among the Tetralophodontidae (*14*, *15*). Given the FAD of Elephantidae (in Africa, some time before 7 Ma), African *Tetralophodon* (13-9 Ma), on not only morphological but also chronological and geographical grounds, is here treated as representative of the elephantid stem group (*83, 84, 85*). It is also the only available tetralophodont at that date – *Anancus* only entered Africa from Eurasia concurrently with appearance in Africa of the earliest known elephants, ca. 7 Ma.

Recent research suggests that the species *Elephas recki* Dietrich, 1915, with a series of chronosubspecies defined by Maglio (*30*) and Beden (*47*), may represent different lineages (*86*). We have therefore named the early 'subspecies' as follows: *"Elephas" brumpti*, *"Elephas" shungurensis*, *Elephas atavus*. Later subspecies of *recki* are accepted to form a monophyletic group with Eurasian 'straight-tusked elephants', for which we accept the generic name *Palaeoloxodon* Matsumoto 1924 (*82, 87*); these are hence named as follows: *Palaeoloxodon recki ileretensis, Palaeoloxodon recki recki*. The terminal member of the series in Africa has been named as a separate species, *Palaeoloxodon jolensis* Pomel, 1895 (syn. *iolensis*).

Relationships among these and the other derived elephantids (*Loxodonta* and *Mammuthus* spp.) is the subject of current revision (*86*). *Palaeoloxodon* has been considered closely related to (or subsumed under) *Elephas* (e.g. *15, 82*), but molecular evidence has suggested a relationship to *Loxodonta* instead (*88, 89*). Morphological data meanwhile have placed *Mammuthus* as sister-group to *Elephas s.s.* and *Palaeoloxodon* as sister to both (*82, 86*). Finally, *Loxodonta* is considered to comprise two distinct lineages, *L. adaurora* on the one hand, and *L. cookei*, *L. exoptata* and *L. africana/cyclotis* on the other (*15*).

The species identifications in our present study are mainly based on Sanders et al. (*15*) and Bobe et al. (*68*), taking into account the recent revision of "*Elephas" recki* group (e.g. *86*) (see further taxonomic references in Table S2). In the case of the genus *Loxodonta* from the Late Miocene (including *L.* sp. from Lukeino), we combined their records under the species name *L. cookei*, due to approximate morphometric similarity of their molars and (at least partial) synonymy with *L. cookei* (*15*).

## 2. Molar morphology

The basic morphology of third molars among the "**gomphothere**" families (Fig. 3e & Extended Data Fig. 9b) is buno-lophodont, where pairs of principal cusps are arranged, often together with smaller conelets, in transverse rows, forming elongate cutting or shearing structures called lophs (in upper teeth) or lophids (in lower teeth), from now on collectively referred to as "lophs". When worn, dentine valleys develop within the lophs, starting from cusp tips. **Mammutids** have zygolophodont molars where the transverse cusp pairs and median conules between them are arranged in strongly tapering, sharp-edged lophs. Tooth wear in

mammutids produces wear facets on the lophs first, leading to a “collapse” of the loph crest after which a dentine valley develops. **Deinotheres** (Extended Data Fig. 9c) are characterized by simple bilophodont molars with two transverse lophs in second and third molars, and similarly to the mammutids, wear facets in the enamel dominate the early stages of tooth wear, dentine valleys developing later following the opening of dentine at the top of the lophs. **Elephantids** (Fig. 3e and Extended Data Fig. 9a) have a derived dental morphology where multiple conelets in transverse rows have become fused to form narrow lophs termed lamellae, that wear into dentine basins surrounded by enamel loops early in tooth wear. These lamellae are ‘cementodont’ - bound together by dental cement that encases the whole tooth crown in derived elephants. These dental modifications are associated with the development of a propalineal (‘proal’) chewing cycle where the jaw moves in antero-posterior direction. During the evolution of the Elephantidae, the number of lamellae increased (horizodonty), the lamellae became antero-posteriorly narrower and increasingly tightly packed, enamel became thinner and hypsodonty increased. **Stegodonts** are a derived family of elephantoid proboscideans that originated in Asia, dispersing to Africa only in the Late Miocene. Stegodont molars evolved convergently to those of elephants (also following a shift to proal mastication (*2*)), but they never reached the highly hypsodont and cementodont molar morphology of derived elephants.

## 3. Supplementary Analyses

**Supplementary Table 1.** Durbin-Watson test (two-sided) results for dental traits with mesowear, core 722 dust data, core 659 dust data and locality mean hypsodonty as independent variables, for the model including all elephantoids during 26 Ma. None of the tests indicate statistically significant autoregression (i.e. autocorrelation of residuals) in these models.

| **variable** | **DW** | **p** |
|---|---|---|
| Hypsodonty | 2.0404 | 0.3407 |
| Loph count | 2.3771 | 0.6767 |
| Loph distance / width | 2.2318 | 0.5315 |
| Enamel thickness | 1.918 | 0.2357 |
| Plicae frequency | 2.3006 | 0.6016 |

**Supplementary Table 2.** Ordinary least squares linear models of the effect of mesowear, LocHyp and core 722 and 659 aeolian dust accumulation data on dental traits. Autoregression of residuals has been tested for by Durbin-Watson tests, and not found for any of the linear models (SupplementaryTable 1). Statistically significant correlations are emboldened. The p-values for overall model fit are based on one-sided F-tests. $R^2$adj values adjusted with the number of terms in the model are shown for overall model fit. All dental traits are significantly related to locality mean hypsodonty values (aridity proxy). Enamel thickness is significantly negatively related to locality mean hypsodonty values and core 722 dust accumulation data.

| All Elephantoidea, 7 Ma - present | | | | | | |
|---|---|---|---|---|---|---|
| Dental trait | Parameter | Estimate | Std. Error | t ratio | p | Summary of model |
| **Hypsodonty** (n. obs. = 38) | (Intercept) | -166.48 | 62.73 | -2.65 | 0.01 | $R^2$adj = 0.73; p < 0.0001 |
| | Mesowear | 0.62 | 0.65 | 0.94 | 0.35 | |
| | **Terrigenous dust (core 722)** | **3.79** | **1.08** | **3.50** | **0.001** | |
| | Terrigenous dust (core 659) | 13.13 | 20.31 | 0.65 | 0.52 | |
| | **Locality mean ordinated HYP** | **53.86** | **13.84** | **3.89** | **0.0005** | |
| **Loph count** (n. obs. = 31) | (Intercept) | -12.28 | 9.46 | -1.30 | 0.21 | $R^2$adj = 0.64; p < 0.0001 |
| | Mesowear | 0.04 | 0.09 | 0.48 | 0.64 | |
| | **Terrigenous dust (core 722)** | **0.47** | **0.12** | **3.88** | **0.0006** | |
| | Terrigenous dust (core 659) | 0.46 | 2.88 | 0.16 | 0.87 | |
| | **Locality mean ordinated HYP** | **3.43** | **1.60** | **2.14** | **0.04** | |
| **Relative loph distance (LD/W)** (n. obs. = 33) | (Intercept) | 0.19 | 0.25 | 0.75 | 0.46 | $R^2$adj = 0.41; p = 0.0007 |
| | Mesowear | 0.00 | 0.00 | 1.61 | 0.12 | |
| | **Terrigenous dust (core 722)** | **-0.01** | **0.00** | **-2.87** | **0.008** | |
| | Terrigenous dust (core 659) | 0.09 | 0.07 | 1.21 | 0.24 | |

| | | | | | | |
|---|---|---|---|---|---|---|
| | **Locality mean ordinated HYP** | **-0.09** | **0.04** | **-2.14** | **0.04** | |
| **Enamel thickness** (n. obs. = 38) | (Intercept) | 12.24 | 2.23 | 5.48 | < 0.0001 | $R^2$adj = 0.72; p < 0.0001 |
| | Mesowear | -0.01 | 0.02 | -0.49 | 0.63 | |
| | **Terrigenous dust (core 722)** | **-0.15** | **0.04** | **-3.84** | **0.0005** | |
| | **Terrigenous dust (core 659)** | **-1.68** | **0.73** | **-2.30** | **0.03** | |
| | **Locality mean ordinated HYP** | **-1.32** | **0.49** | **-2.69** | **0.01** | |
| **Plicae frequency** (n. obs. = 35) | (Intercept) | -20.99 | 6.39 | -3.28 | 0.003 | $R^2$adj = 0.50; p < 0.0001 |
| | **Mesowear** | **0.13** | **0.06** | **2.06** | **0.048** | |
| | **Terrigenous dust (core 722)** | **0.22** | **0.10** | **2.12** | **0.04** | |
| | Terrigenous dust (core 659) | 2.86 | 2.18 | 1.31 | 0.20 | |
| | Locality mean ordinated HYP | 0.60 | 1.26 | 0.48 | 0.64 | |
| **Elephantidae, 7 Ma - present** | | | | | | |
| Dental trait | Parameter | Estimate | Std. Error | t ratio | p | Summary of model |
| **Hypsodonty** (n. obs. = 31) | (Intercept) | -160.77 | 88.61 | -1.81 | 0.08 | $R^2$adj = 0.70; p < 0.0001 |
| | Mesowear | 0.54 | 0.87 | 0.62 | 0.54 | |
| | **Terrigenous dust (core 722)** | **4.09** | **1.24** | **3.29** | **0.003** | |

| | | | | | | |
|---|---|---|---|---|---|---|
| | Terrigenous dust (core 659) | 17.24 | 29.32 | 0.59 | 0.56 | |
| | **Locality mean ordinated HYP** | **50.76** | **16.61** | **3.06** | **0.005** | |
| **Loph count** (n. obs. = 26) | (Intercept) | -8.36 | 9.58 | -0.87 | 0.39 | $R^2$adj = 0.63; p < 0.0001 |
| | Mesowear | 0.02 | 0.09 | 0.24 | 0.82 | |
| | **Terrigenous dust (core 722)** | **0.42** | **0.12** | **3.54** | **0.002** | |
| | Terrigenous dust (core 659) | 1.22 | 2.88 | 0.42 | 0.68 | |
| | **Locality mean ordinated HYP** | **3.19** | **1.59** | **2.01** | **0.057** | |
| **Relative loph distance (LD/W)** (n. obs. = 27) | (Intercept) | 0.32 | 0.21 | 1.51 | 0.15 | $R^2$adj = 0.38; p = 0.005 |
| | Mesowear | 0.00 | 0.00 | 0.86 | 0.40 | |
| | **Terrigenous dust (core 722)** | **-0.01** | **0.00** | **-2.29** | **0.03** | |
| | Terrigenous dust (core 659) | 0.05 | 0.07 | 0.81 | 0.428 | |
| | Locality mean ordinated HYP | -0.07 | 0.03 | -1.93 | 0.067 | |
| **Enamel thickness** (n. obs. = 31) | (Intercept) | 13.37 | 2.65 | 5.05 | <0.0001 | $R^2$adj = 0.73; p < 0.0001 |
| | Mesowear | -0.03 | 0.03 | -1.05 | 0.3029 | |
| | **Terrigenous dust (core 722)** | **-0.11** | **0.04** | **-2.92** | **0.0071** | |

| | | | | | | |
|---|---|---|---|---|---|---|
| | **Terrigenous dust (core 659)** | **-1.82** | **0.88** | **-2.08** | **0.0475** | |
| | **Locality mean ordinated HYP** | **-1.42** | **0.50** | **-2.85** | **0.0084** | |
| **Plicae frequency (n. obs. = 29)** | (Intercept) | -23.92 | 7.31 | -3.27 | 0.003 | $R^2$adj = 0.53; p = 0.0001 |
| | **Mesowear** | **0.15** | **0.07** | **2.09** | **0.048** | |
| | **Terrigenous dust (core 722)** | **0.24** | **0.11** | **2.11** | **0.046** | |
| | Terrigenous dust (core 659) | 1.36 | 2.65 | 0.51 | 0.61 | |
| | Locality mean ordinated HYP | 1.32 | 1.45 | 0.91 | 0.37 | |
| **All Elephantoidea, 26 Ma - present** | | | | | | |
| Dental trait | Parameter | Estimate | Std. Error | t ratio | p | Summary of model |
| **Hypsodonty** (n. obs. = 57) | (Intercept) | -109.53 | 53.42 | -2.05 | 0.05 | $R^2$adj = 0.56; p < 0.0001 |
| | **Mesowear** | **1.12** | **0.53** | **2.10** | **0.04** | |
| | Terrigenous dust (core 659) | 4.32 | 17.41 | 0.25 | 0.80 | |
| | **Locality mean ordinated HYP** | **43.37** | **9.66** | **4.49** | **<0.0001** | |
| **Loph count** (n. obs. = 49) | (Intercept) | -9.74 | 5.82 | -1.67 | 0.10 | $R^2$adj = 0.71; p < 0.0001 |
| | Mesowear | 0.06 | 0.06 | 0.98 | 0.33 | |
| | Terrigenous dust (core 659) | 2.17 | 1.81 | 1.2 | 0.24 | |

| | | | | | | |
|---|---|---|---|---|---|---|
| | **Locality mean ordinated HYP** | **5.83** | **1.04** | **5.62** | **<0.0001** | |
| **Relative loph distance (LD/W)** (n. obs. = 50) | (Intercept) | 0.61 | 0.18 | 3.38 | 0.002 | $R^2$adj = 0.67; p < 0.0001 |
| | Mesowear | 0.00 | 0.00 | 0.6 | 0.55 | |
| | Terrigenous dust (core 659) | 0.00 | 0.06 | -0.03 | 0.97 | |
| | **Locality mean ordinated HYP** | **-0.20** | **0.03** | **-6.54** | **<0.0001** | |
| **Enamel thickness** (n. obs. = 51) | (Intercept) | 15.19 | 2.11 | 7.2 | <.0001 | $R^2$adj = 0.64; p < 0.0001 |
| | **Mesowear** | **-0.07** | **0.02** | **-3.39** | **0.001** | |
| | **Terrigenous dust (core 659)** | **-2.23** | **0.70** | **-3.19** | **0.003** | |
| | **Locality mean ordinated HYP** | **-1.00** | **0.36** | **-2.73** | **0.009** | |
| **Plicae frequency** (n. obs. = 51) | (Intercept) | -14.20 | 4.22 | -3.36 | 0.002 | $R^2$adj = 0.44; p < 0.0001 |
| | **Mesowear** | **0.11** | **0.04** | **2.64** | **0.01** | |
| | Terrigenous dust (core 659) | 2.03 | 1.46 | 1.38 | 0.17 | |
| | Locality mean ordinated HYP | 1.27 | 0.75 | 1.68 | 0.10 | |

**Supplementary Table 3.** Results of Multiple Regression Commonality Analyses; the numbers give percent variance explained. Emboldened values indicate largest percentage of variance explained for each dental trait. **Above**, with mean mesowear angle of all elephantoids as the dependent variable, and Terrigenous (aeolian) sediments from (ODP core 659, ‘Terrig’) and estimated grass percentage of East African environments (‘Grass’) as predictors. The positive correlation to grass explains the majority of uniquely explained variation in each case. **Below**, with dental traits of all elephantids as dependent variable and aridity proxies (locality mean ordinated hypsodonty and terrigenous sediments (ODP core 722, ‘Terrig’,

respectively) and dental mesowear (dietary proxy) as independent (predictor) variables. Analyses using dust data from both raw data and data with temporal trend removed from the time series are shown.

| **Dental trait** | **Variables** | | |
|---|---|---|---|
| **Dental trait** | **Variables** | **All Elephantoidea, 26 Ma - present** | |
| | | **Locality means** | **Species means** |
| **Mesowear** | Unique to LocHyp | 12.4 | 10.3 |
| | Unique to Terrig (core 659) | 1.1 | 2.8 |
| | **Unique to Grass** | **24.3** | **16.5** |
| | Common to LocHyp & Terrig (core 659) | -0.7 | -2.4 |
| | Common to & LocHyp Grass | -1.6 | -1.7 |
| | Common to and Terrig (core 659) & Grass | -4.1 | -4.3 |
| | **Common to LocHyp, Terrig (core 659) & Grass** | **38.6** | **26.2** |
| | Total | 69.9 | 47.4 |
| | | **Elephantidae 5 Ma** | |
| | | **with trend** | **detrended** |
| **Hypsodonty** | **Unique to LocHyp** | **50.6** | **1.4** |
| | Unique to Mesowear | 2.6 | 0.6 |
| | Common to LocHyp & Mesowear | 4.1 | -0.6 |
| | Total | 57.3 | 1.4 |
| **Loph count** | **Unique to LocHyp** | **28.5** | **3.2** |
| | Unique to Mesowear | 8.3 | 0.6 |
| | Common to LocHyp & Mesowear | 6.2 | -0.3 |
| | Total | 43.0 | 3.6 |
| **Loph distance / molar width** | **Unique to LocHyp** | **31.2** | **9.2** |
| | Unique to Mesowear | 0.1 | **8.7** |
| | Common to LocHyp & Mesowear | 3.5 | -7.2 |

| | | | |
|---|---|---|---|
| | Total | 34.8 | 10.7 |
| **Enamel thickness** | Unique to LocHyp | 50.1 | -13.4 |
| | Unique to Mesowear | 7.6 | 4.4 |
| | **Common to LocHyp & Mesowear** | **11.9** | **14.2** |
| | Total | 69.7 | 5.2 |
| **Plicae frequency** | **Unique to LocHyp** | **34.0** | **16.6** |
| | **Unique to Mesowear** | **23.8** | **18.5** |
| | Common to LocHyp & Mesowear | -11.0 | -16.5 |
| | Total | 46.7 | 18.6 |
| | | **Elephantidae 5 Ma** | |
| | | **with trend** | **detrended** |
| **Hypsodonty** | **Unique to Terrig** | **39.7** | 0.3 |
| | Unique to Mesowear | 0.9 | 0.0 |
| | Common to LocHyp & Mesowear | 5.8 | 0.0 |
| | Total | 46.3 | 0.3 |
| **Loph count** | **Unique to Terrig** | **39.0** | **9.0** |
| | Unique to Mesowear | 1.7 | 2.9 |
| | Common to LocHyp & Mesowear | 12.9 | -2.6 |
| | Total | 53.6 | 9.3 |
| **Loph distance / molar width** | **Unique to Terrig** | **22.1** | 0.0 |
| | Unique to Mesowear | 0.1 | 1.4 |
| | Common to LocHyp & Mesowear | 3.5 | 0.2 |
| | Total | 25.7 | 1.5 |
| **Enamel thickness** | **Unique to Terrig** | **32.4** | 0.6 |
| | **Unique to Mesowear** | 7.5 | **18.7** |
| | Common to LocHyp & Mesowear | 12.1 | -0.1 |
| | Total | 52.0 | 19.1 |
| **Plicae frequency** | **Unique to Terrig** | **29.5** | **3.0** |

| | | | |
|---|---|---|---|
| | Unique to Mesowear | 3.6 | 0.5 |
| | Common to LocHyp & Mesowear | 9.2 | 1.5 |
| | Total | 42.3 | 5.1 |

**Supplementary Table 4.** One-way univariate significance tests (t-tests) demonstrating hypsodonty increase from *Stegotetrabelodon orbus* and *Primelephas korotorensis* to *Loxodonta cookei*, and loph increase from all three to *Loxodonta adaurora.* Each species sample is combined across localities where it occurs.

| | **t** | **p** | **df** |
|---|---|---|---|
| **Hypsodonty** | | | |
| *cookei-orbus* | 4.321 | 0.0015 | 10 |
| *cookei-korotorensis* | 3.335 | 0.003 | 22 |
| **Loph count** | | | |
| *adaurora-orbus* | 7.605 | 0.000001 | 16 |
| *adaurora-korotorensis* | 7.393 | <0.000001 | 28 |
| *adaurora-cookei* | 3.098 | 0.008 | 14 |

## 4. Regional changes in vegetation and proboscidean diets in East Africa during the Neogene

Changes of proboscidean diets in East Africa during the Neogene have previously been largely analysed based on stable carbon isotopes of dental enamel (*21, 77, 90*), which indicate a universal dietary shift from C3-dominated to C4-dominated diets in elephantids and *Anancus* during the Late Miocene (ca. 8 Ma) (*1, 77*). However, this observation is largely based on data from Turkana Basin in Kenya, while other regions during the Late Miocene in East Africa have not been as thoroughly analysed. Indeed, stable isotope evidence from Tugen Hills indicates that a shift to C4 diet only happened in *Anancus*, not in early elephants there (*91*). Moreover, while the shift to C4 diets largely indicates a shift to grazing on C4 grasses, the stable carbon isotope data do not capture feeding on C3 grasses.

We demonstrate that the shift of early elephants to grazing during the Late Miocene happened in Turkana specifically (Lothagam, Nawata Formation), while other palaeopopulations of early elephants in Tugen Hills

and Samburu Hills, Kenya, Nkondo Formation in Uganda and Manonga Valley in Tanzania retained browse-dominated diets (Fig. 2; Fig. S2). Moreover, these regional differences in early elephant diets follow differences in vegetation structure: while palaeobotanical proxies indicate forest-dominated paleoenvironment in Nkondo, Uganda, and woodland in Tugen Hills, the estimated grass cover in Nawata FM, Turkana, is as high as ca. 50 % indicating a grassy woodland or wooded grassland savanna (Fig. 2; Section 4 below). As suggested by Fortelius et al. (*42*), the Turkana region may have been special in terms of early aridification (during the Late Miocene), and consequent adaptation of mammals to grazing. The high estimated C4 grass proportion in the Nawata FM in the Late Miocene, and the corresponding grazing mesowear signal in early elephants there while surrounding regions were still dominated by proboscideans with browse-dominated diets, lends support for this hypothesis (Fig. S4). Since the Late Miocene, a consistantly high proportion of C4 grasses occurred in the Turkana region, while surrounding areas also became dominated by grasslands and grazing proboscideans, especially during the Pleistocene (Section 4 below; Extended Data Fig. 5).

## 5. Testing for correlated evolution of proal chewing and major steps in the dental trait evolution of Proboscidea

We performed a Pagel's test (*66*) in order to test whether the major evolutionary changes in loph count ("horizodonty") and hypsodonty of molars were correlated with the evolution of proal (fore-aft) chewing cycle in proboscideans. For this purpose we used a strict consensus supertree of Proboscidea based on 300 trees used by Cantalapiedra et al. (*58*). Only binary trait stages are possible to test using the Pagel's test, so for the purpose of our analyses, we coded the presence/absence of 1) proal chewing, 2) last molar height/width ratio more than 1.2 (i.e. having hypsodont molars), and 3) six or more lophids/lamellae in the last molar (i.e. having markedly increased horizodonty). We defined "markedly increased horizodonty" as having 6 or more lophids/lamellae, because having 5 or less lophids is the most common condition for non-elephantid proboscideans and an increase beyond that can be regarded a derived condition in this regard (see also Cantalapiedra et al. (*58*)). These traits were coded for all of the 185 proboscidean taxa identified in the supertree of Cantalapiedra et al. (*58*). We then used R package "phytools" in R Studio version 4.2.2 to perform Pagel's tests for the following trait pairs: 1) proal chewing – hypsodont molars, and 2) proal chewing – marked horizodonty.

Our results indicate that increase in loph count into 6 or more in Proboscidea was phylogenetically correlated with the evolution of proal chewing, whereas significant direct phylogenetic correlation between proal chewing and hypsodonty is not supported, as shown by the AIC and p-values for the proal-hypsodont and proal-horizodont dependent models (Supplementary Table 1).

**Supplementary Table 5**. Pagel's test results for proal-hypsodont (left) and proal-horizodont (right) model comparisons.

| proal (x)., hypsodont (y) | | | | | proal (x)., horizodont (z) | | | | |
|---|---|---|---|---|---|---|---|---|---|
| **Independent model rate matrix** | | | | | **Independent model rate matrix** | | | | |
| | 0\|0 | 0\|1 | 1\|0 | 1\|1 | | 0\|0 | 0\|1 | 1\|0 | 1\|1 |
| 0\|0 | -0.38 | 0.20 | 0.17 | 0.00 | 0\|0 | -0.95 | 0.77 | 0.17 | 0.00 |
| 0\|1 | 8.77 | -8.95 | 0.00 | 0.17 | 0\|1 | 1.52 | -1.69 | 0.00 | 0.17 |
| 1\|0 | 0.60 | 0.00 | -0.81 | 0.20 | 1\|0 | 0.60 | 0.00 | -1.38 | 0.77 |
| 1\|1 | 0.00 | 0.60 | 8.77 | -9.37 | 1\|1 | 0.00 | 0.60 | 1.52 | -2.12 |
| **Dependent (x & y) model rate matrix** | | | | | **Dependent (x & z) model rate matrix** | | | | |
| | 0\|0 | 0\|1 | 1\|0 | 1\|1 | | 0\|0 | 0\|1 | 1\|0 | 1\|1 |
| 0\|0 | -0.17 | 0.00 | 0.17 | 0.00 | 0\|0 | -2.92 | 2.92 | 0.00 | 0.00 |
| 0\|1 | 8.07 | -8.07 | 0.00 | 0.00 | 0\|1 | 37.79 | -40.27 | 0.00 | 2.48 |
| 1\|0 | 0.62 | 0.00 | -2.29 | 1.68 | 1\|0 | 8.31 | 0.00 | -8.31 | 0.00 |
| 1\|1 | 0.00 | 0.00 | 0.00 | 0.00 | 1\|1 | 0.00 | 0.65 | 0.00 | -0.65 |
| **Model fit** | | | | | **Model fit** | | | | |
| | log-likelihood | AIC | | | | log-likelihood | AIC | | |
| independent | -47.90 | 103.80 | | | independent | -77.26 | 162.53 | | |
| dependent | -44.46 | 104.91 | | | dependent | -55.28 | 126.56 | | |
| **Hypothesis test result** | | | | | **Hypothesis test result** | | | | |
| likelihood-ratio | 6.88 | | | | likelihood-ratio | 43.97 | | | |
| p-value | 0.14 | | | | p-value | 0.0000000065 | | | |

Moreover, our results suggest that a model where increase in loph count into 6 or more depends on the evolution of proal chewing, rather than vice versa, is the most supported model of the relationship between these two traits, as indicated by AIC and AIC weight values (Supplementary Table 2). This finding supports the suggestion of Saegusa (*2*) that the evolution of proal chewing in Proboscidea was a "key adaptation" that facilitated increase in loph count, leading to a major increase in shearing efficiency. As our new results indicate (in concert with Saegusa (*2*)), this trait combination evolved independent of adaptation to grazing and was at least in the case of East African proboscideans associated with the aridification of climate. Our results also suggest that the increase in hypsodonty is a further adaptation to increase functional durability in response to increases in aridity, but it is not directly correlated with the evolution of proal chewing and increase in loph count.

**Supplementary Table 6.** AIC and AIC weight comparisons for the models where proal chewing and increase in loph count into 6 or more (horizodonty) are 1) independent from each other, 2) proal chewing depends of horizodonty, 3) horizodonty depends on proal chewing and 4) proal chewing and horizodonty depend on each other.

| **Proal (x) - Horizodonty (z) Pagel's test model comparisons** | | | | |
|---|---|---|---|---|
| | 1.independent | 2.dependent x | 3.dependent z | 4.dependent x&z |
| AIC | 162.53 | 134.21 | 124.13 | 126.56 |
| AIC weights | 0.00 | 0.00 | 0.77 | 0.23 |

**A note on causality**

Our analyses show correlations between diet, aridity and the evolution of dental traits, and causal relationships were argued based on the temporal correlation between events or order in which these changes happened (e.g., the 'proal' chewing mechanism preceded changes in dental traits and, later, peaks in aridity were coincident with or slightly preceded successive increases in loph count and hypsodonty). Nonetheless, inferring causality from correlation is risky, and to address this concern we applied Pagel's Test of phylogenetically correlated evolution of traits (*66*) to test Saegusa's hypothesis of proal chewing as a key adaptation permittive of the evolution of other dental traits. In this we used the proboscidean supertree published by Cantalapiedra et al. (*58*). Our results (Suppl. 4) support a model where increase in loph count (to 6 or more) in derived proboscideans was indeed dependent on the evolution of proal chewing. This model had higher likelihood than ones where these traits were independent, depended on each other, or proal chewing depended on loph count.

This result supports the idea that the increase in loph count was causally related to major changes in the masticatory system into a proal chewing cycle, so the latter was, as Saegusa and others have supposed, a "key innovation" allowing improvement in molar shearing efficiency to deal with vegetational changes brought by aridification. However, the results do not provide support for the evolution of hypsodonty being also consequent on proal chewing, at least not directly; it was rather a later adaptation to increase functional durability of the molars.

Our second conclusion on causality concerned the correlation between later trends in the dental traits and dust flux (aridity proxy). We quantitatively tested this temporal relationship using breakpoint analysis (Fig. 4), that shows clear temporal correlation between peaks in dust flux and increments in dental traits. We considered further methods that compare time-series of dependent and independent variables, but it would be difficult to apply these to our data because the time-series of dental traits have unavoidable gaps resulting from the nature of the fossil record. In any case, by the very nature of the ratchet pattern we uncovered, these methods would fail because the curves are not expected to match perfectly; only *increases* in the independent variable drive changes in the dependent one; this is why we employed breakpoint analysis.

As far as inferring causality from this correlation, there are three possibilities: (i) aridity drove dental evolution, (ii) dental evolution drove aridity, (iii) aridity and dental evolution were driven by a third factor. Option (ii) can be discounted. Option (iii) is possible but we do not impute the dust proxy as necessarily the causal factor, but aridity more generally - a deliberately high-level environmental factor - and it is hard to envisage what at a higher level still could have independently impacted dental evolution of terrestrial mammals as well as triggering arid climate. We therefore argue that the pattern of stepwise increase in hypsodonty and loph count that clearly correlates to phases of increased dust flux suggests a causal relationship from some aspect of aridification to the evolution of these dental traits.

Finally, our data for diet and dental traits of proboscideans includes a spatial pattern in addition to a temporal one. Our combination of time series and locality-based analyses support the hypothesis of causal relationship of aridification and diet on dental evolution, as we first see major changes in dental traits in localities that indicate locally more arid conditions than in other contemporaneous parts of East Africa (for example, in the Turkana region in Late Miocene), and these also tend to be areas with the first evidence of expansion of grass-rich plant communities and grazing diets.

## 6. Published data on East African Neogene palaeoenvironments

EARLY MIOCENE

- Rusinga (17.8 Ma):
    - Wet lowland forest (*92*)
    - Closed forest/woodland (*93*):
        - The macrofossil flora is dominated by a large and diverse flora of trees, shrubs and climbing plants; no grasses were recorded (although they might have been present (*94*))
        - No *Acacia* trees
        - Mixture of woodland plants (similar to those found today around Lake Victoria) and East African forest plants
        - Predominantly woodland but patches of forest with large trees occurred, and this is reflected in the fossil fauna
    - Maxbauer et al. (*95*):
        - Plant macrofossils indicate a riparian environment with a patchwork of woodland and forest biomes in a strongly seasonal warm climate
        - Leaf size distribution and lack of grasses are more consistent with predominantly forest than woodland habitats
    - Grossman et al. (*96*):
        - Hiwegi: Numerous localities but forest conditions at least in some – evidence for more open conditions
        - Kulu: Lake and a forest – perhaps drier and more open than Hiwegi
    - Uno et al. (*97*): a minor element of ca. 15 % C4 biomass may have been present in Hiwegi, as estimated from $\delta^{13}C$ of organic soil matter
    - Peppe et al. (*24*): $\delta^{13}C$ values from soil organic matter from the Hiwegi Formation (where most of the proboscidean fossils come from) are mostly consistent with forest environments. Soil carbonate $\delta^{13}C$ could only be analysed from Kiahera FM, where it indicates significant proportion of C4 photosynthesising vegetation. One sample from R3, Hiwegi FM, contained significant amount of C4 grass phytoliths.
- Karungu (17.8 Ma)
    - Forest/woodland with low grass % (0 to 2 %), as indicated by phytolith analysis (*23*)
    - Seasonal, partly open woodland with locally abundant C4 grasses, based on soil $\delta^{13}C$ and phytolith analyses (*24*)
- Moroto, Uganda (20.6 Ma)
    - Thick forest with grassy spaces (*96*)

- Grass comprised ca. 30 – 55 % of the vegetation (based on phytolith analysis), resulting in a mean of ca. 42 % (*23*)
- Seasonal woodland with C4 grasses present, based on soil $\delta^{13}C$ and phytolith analyses (*24*)

- Meswa Bridge (22.5 Ma)
    - Tassy and Pickford (*98*) (based on mollusc fauna): relatively dry and open forest, with minimum annual precipitation of ca. 900 mm
- Bukwa, Uganda (19 Ma)
    - Chaney (*99*); Hamilton (*100*) (as referenced in (*101*)):
        - "in situ plants, grass culms and silicified woods"…"that represent Sudano-Zambesian Miombo-like woodlands"
    - Grassland paleoenvironment, based on mollusc fauna (*102*)
    - Early grassland-dominated environment; phytolith data indicate grass % as high as 36 – 92 % of vegetation, with a mean of 64 % (*23*)
    - Seasonal woodland with C4 grasses present, based on soil $\delta^{13}C$ and phytolith analyses (*24*)
- Koru (19 – 20 Ma)
    - Forest (*96*)
    - 0 to 29 % grass in the phytolith data, with a mean of ca. 15 % (*23*)
- Napak (ca. 20 Ma)
    - Forest – some grassy and more open areas may be present (*96*)
    - Forest environments with very low grass % (4-7 %), based on phytolith analysis (*23*)
    - Predominantly forested paleoenvironment based on soil $\delta^{13}C$ and phytolith analyses (*24*)
- Songhor (19 – 20 Ma)
    - Montane rain forest – may have some grassy more open areas present (*96*)
    - 7-8 % grass in the phytolith data (*23*)
- Kalodirr & Moruorot (16.8 – 17.5 Ma)
    - Tropical woodland, seasonal, meandering streams (*96*)
    - Grass % in phytolith samples ranges from 0 to 17 %, with a mean of ca. 10 % (*23*)
- Buluk (ca. 17.2)
    - Deciduous forest – tropical woodland, seasonal, fluvial (*96*)
    - Woodland with prominent grassy areas (5 – 33 % grasses, mean of ca. 18 %), as revealed by phytolith analysis (*23*)
    - Seasonal woodland with C4 grasses present, based on soil $\delta^{13}C$ and phytolith analyses (*24*)

MIDDLE MIOCENE

- Nachola (Aka Aiteputh, ca. 15.5 Ma)
  - Kunimatsu et al. (*103*)
    - The presence of *Mioeuoticus* supports forest-dominated paleoenvironment
    - Forested palaeoenvironment suggested by plentiful petrified wood (*104*)
    - Brachydont, browser-dominated ungulate fauna, further supporting predominantly forested paleoenvironment (*105*)
- Maboko (ca. 15 Ma)
  - Riparian woodland, nyika bushland, seasonally wet wooded grassland (*96*)
  - Cenogram data of mammals indicates a mosaic of woodland and open grassland savanna largely similar to today's East Africa (*106*)
  - Presence of C3 grasses and possibly CAM plants undermines the utility of isotope data for revealing the presence of grasslands (*7*, *107-109*)
  - Retallack et al. (*109*):
    - Paleosols show evidence of seasonal waterlogging as expected in a lowland fluvial basin (also *110*)
    - Root traces and stump casts indicate the presence of trees at least periodically
    - Indications of dry (but not desert) climate in paleosols
    - Seasonal variation between dry and waterlogged
    - Analogous soils today e.g. near the Tsavo River near Ngulia, Tsavo National Park (although more deeply weathered red soil)
    - Fossil flora from laterally equivalent Nyakach Formation at Kaimogool indicates dry conditions too (*111*)
    - Fossil land snails from Maboko represent a fauna similar to nyika bushland and gallery woodlands of today's Tana River with mean annual precipitation of < 500 mm and hot climate
    - Palaeovegetation interpretations:
      - Yom palaeosols: arid, seasonally waterlogged wooded grassland similar to modern dambo or vlei in Africa
      - Dhero palaeosols: woodland in early ecological succession at stream margins
      - Mogo palaeosols: salt scrub of woody bushes with little herbaceous cover of ground with seasonal salt efflorescences

            - Ratong palaeosols: dry shrubland and woodland (nyika) similar as e.g. today in the distal levee of Tsavo River near Ngulia, Tsavo National park
        - Mammal fauna:
            - Numerous fossils excavated from Yom and Dhero paleosols
            - *Afrochoerodon* is found from Yom pedotype (indicating dambo wooded grassland environment), *Protanancus* and *Prodeinotherium* mostly from swale (flood deposit) and Ratong paleosol (indicating nyika bushland vegetation)
    - Arney et al. (112): All the fossil large herbivorous mammals at Maboko had C3-plant based diets, as indicated by enamel $\delta^{13}C$ isotope analyses.
- Nyakach Formation (15 Ma) (*111*):
    - Similar to Maboko, part of the same stratigraphic unit containing similar paleosols
    - Plant macrofossils:
        - Different from those of Rusinga palaeoflora: leaf fossils are smaller, less diverse and more coriaceous, and fossil grasses are present
        - Vegetation was like that of modern Kenyan and Tanzanian *Acacia-Gommiphora* wooded grassland
        - "the most ancient known grass-rich assemblage with small-leaved grassland dicots in East Africa, predating the better known grass-dominated flora at Fort Ternan" (but see Bukwa and Moroto, Early Miocene)
- Kipsaraman (Tugen Hills)
    - Forested palaeoenvironment, as opposed to the more open Maboko (*113*)
    - ''poorly preserved leaves and stems [. . . and] small fragments of silicified wood [. . .]'' (*114, 115*)
    - C4 grasses were present in the Muruyur Beds; paleosol carbonate $\delta^{13}C$ between -7.4 and -5.2; average: -6.3, which corresponds with ca. 40 % C4 biomass (*97*)
- Fort Ternan (ca. 13.8 Ma)
    - Woodland/grassland (*116-120*)
    - Palynological analysis and paleosols (*117*)
        - Grass pollen comprises 54 % of the total pollen count
    - Jacobs (*94*): "A single pollen assemblage from Fort Ternan contains 54% grass pollen, aquatics and montane taxa, indicating an open woodland, with montane species in nearby highlands" (*117*).
    - Retallack (*119*):
        - *Acacia-Commiphora* wooded grassland

        - Grasses of the subfamily Chloridoideae and the supertribe Panicanae were common in tropical Africa already in Middle Miocene, but there is no evidence of grasses of the supertribe Andropogonae which are now dominant in the seasonally arid, overgrazed and burned African grasslands
        - “The suprageneric mix of fossil grasses at Fort Ternan is unlike that of a fire-prone, overgrazed, or otherwise disturbed habitat. Nor is it like an open grassland. The proportion of Chloridoideae to Panicanae in the Fort Ternan assemblage is compatible with interpretation as a wooded grassland, grassy woodland, or seasonally waterlogged ("dambo" or "vlei") grassland. Especially similar to the Fort Ternan assemblage in suprageneric composition are *Eragrostis* grasslands of seasonally flooded pans in a region of Zambezian dry woodland in Zimbabwe (map unit E6 of Rattray, 1960), but these differ in having also an arundinoid component (*Phragmites*).”
        - Isotopes indicate less than 10 % C4 grasses, although most of the modern relatives of Fort Ternan fossil grasses are C4 photosynthesising (*120*)
- Ngorora Formation, Members A – C (13 – 11 Ma)
    - Jacobs and Kabuye (*121*):
        - 13 – 11 Ma (Kabarsero): Rainforest vegetation, with ca. 15 % herbs; grass is rare and comprises only rainforest grasses (*Leptaspis* and *Humbertochloa*)
    - Jacobs and Winkler (*122*):
        - 13 – 11 Ma (Kabarsero): three macrofossil samples from block B, of which two had no grass and one had ca. 6.7 % grass
    - Rasmussen et al. (*123*):
        - Grass pollen present in Ngorora Members B and C: semi-quantitative pollen analysis result indicates ca. 14.3 % grass in the sample from Kelonechkun 2 (Member B), and ca. 33.3 % grass in Bossei 1 (Member C)
        - “The older part of the Ngorora Formation (c. 13.3–12 Ma) records low-energy settings of lakes, floodplains and palaeosols, and evidence of analcime indicates that lakes were alkaline. The palynomorph spectrum consists of tree pollen (*Juniperus*, *Podocarpus*), Euphorbiaceae pollen (*Acalypha*, *Croton*) and herbaceous pollen of Poaceae and Asteraceae, suggestive of wooded grasslands or grassy woodlands”
        - “Alkaline lakes, floodplains and palaeosols continue upsection (c. 12–9 Ma), but environmental fluctuations become more dynamic. Paucity of palynomorphs and the presence of an equid may point to progressively drier conditions”
        - “As records of grasslands that pre-date late Miocene time are rare, our finding of middle Miocene (12–13 Ma) grassy savannah in the Central Kenya Rift is also relevant to models of human evolution in East Africa.”

LATE MIOCENE

- Samburu Hills, Namurungule Formation
    - Change in climate ca. 9.6 Ma towards wetter, more seasonal conditions (*124*)
    - Based on mammal fauna, the environment was likely a woodland, surrounded by more open environments; footprints indicate presence of swamp (*125*)
    - Ishida (*126*): "The faunae and florae of Samburu Hills and Nachola show that the palaeoenvironment of *Kenyapithecus* might be a thick forest with rich water, but that of the Samburu hominoid woodland-savanna."
- Tugen Hills
    - "The sequence of lowland or submontane forest at 12.6 Ma, seasonally arid woodland or wooded savanna at about 10 Ma, and dry forest to woodland and upland forests at 6.8 Ma among localities that are within 10 km of each other demonstrates that paleoenvironmental change was not unidirectional from forest to savanna but reflected the combined effects of global climate change and regional physiographic development." (*127*)
    - Jacobs (*94*):
        - "Tugen Hills palaeosol isotope records and carbon isotopes from fossil tooth enamel apatite consistently indicate the presence of a C4 component on the landscape and in the mammalian diet as early as 15 Myr ago (*110, 100*). Palaeosol carbonates document mixed C3–C4 vegetation throughout the sequence, but herbivore tooth enamel apatite indicates a shift towards an exclusive C4 diet between 8.5 and 6.5 Myr ago (*128*).
    - Ngorora Formation, Members D – E (10 – 8.5 Ma)
        - C13 isotopes from soil carbonates: ca. -6 = ca. 45 % C4 grasses (*90*)
    - Mpesida Beds (7.2 – 6.2 Ma)
        - Various types of forest (*129, 130* supplement)
        - Plant macrofossils indicate wet lowland forest community, having floral affinities with West and Central Africa, and possibly with Indo-Malaya
        - In some fossil tree stumps, the diameter exceeds 90 cm and those trees would have been at least 50 m tall
        - Mean pedogenic carbonate $\delta^{13}C$: -7.64 = ca. 32 % C4 plants (*129*)
    - Lukeino Formation (ca. 6 Ma)
        - Bamford et al. (*130*):
            - Warm and wet deciduous forest/woodland
            - 38 dicot leaf types, 1 monocot and 2 ferns in plant macrofossil record
            - Mammal fauna also indicates quite closed environment: grazing equids and rhinos are uncommon, most bovids (e.g. *Cephalophus* sp., *Tragelaphus* cf. *spekei*) and all proboscideans and giraffids are brachydont, there is one

        lophodont and one bunodont suid, and *Deinotherium*, chalicotheres and *Hyaemoschus aquaticus* are present

    - Forest – grassy woodland, based on $\delta^{13}C$ of soil carbonates (ca. 20 – 45 % C4 grasses) (*131*)

- Lemudong'o (6 Ma)
  - Paleolake basin at the western margin of eastern Rift Valley, Narok County, South-Western Kenya
  - More humid than Lothagam, "Lukeino has perhaps the greatest geomorphological and ecological similarity to LEM 1" (*132*)
  - "Local environment of permanent gallery-forest near the fluctuating margin of a shallow lake in a small tectonically formed rift-valley basin. More open woodland to wooded grasslands occurred nearby." (*133*)
  - Ambrose et al. (*132*): "Its spectrum of terrestrial habitats resembles that of several penecontemporary fossil sites from the late Miocene of the Gregory Rift Valley in eastern Africa, including the Lukeino Formation in the Baringo Basin of northern Kenya, and the western margin of the Middle Awash Valley, Ethiopia. LEM 1 bears less similarity to other equatorial sites adjacent to large lakes and rivers that contain more arid-adapted terrestrial faunas and diverse aquatic faunas, such as Toros-Menalla in the Lake Chad paleobasin, the Nawata Formation of Lothagam in the Turkana basin, and the Manonga Valley paleobasin in Tanzania."
  - Fauna indicates closed, forested environment:
    - *Ahterurus* -porcupine present, found today only in forest environments (*133*)
    - Suids are represented by *Nyanzachoerus*, which "has been associated with more forested, or closed habitats (*134-135*, *136* for a contrary view)" (*137*)
    - Hyracoids are represented by tree hyraxes (*Dendrohyrax*), which are arboreal and mostly nocturnal, and indicate forested environment (*138*)
      - Small size of the species may indicate dry forest rather than rainforest
    - Rodent fauna is dominated by Murinae relative to Gerbillinae (*139*)
    - Postcranial morphology of cercopithecine primates suggests closed, forested habitat (*140*)
    - Postcranial (astragalus and phalanx) morphology of bovids indicates the presence of forest and/or light cover (*141*)
    - Only the presence of hipparionines (*Eurygnathohippus*) and some rodents indicates the presence of more open habitat, but their low abundance indicates that they represent a more distant open landscape community (*139, 142*)
- Lothagam
  - Mosaic of floodplain savannas dissected by gallery woodlands; regular annual or semi-annual dry seasons throughout the sequence (ca. 9 – 4.2 Ma) (*143*)

- Increased aridity between 6.7 and 5 Ma, based on palaeosols (*143*)
- Grass cover estimates based on $\delta^{13}C$ of soil carbonates (*21* appendix):
  - Lower Nawata (7.4 – 6.4 Ma): grassy woodland – wooded grassland (ca. 20 – 70 % C4 grasses, mean=ca. 48 %)
  - Upper Nawata (6.4 – 5.6 Ma): grassy woodland – wooded grassland (ca. 45 – 78 % C4 grasses, mean=ca. 55 %)
  - Apak (4.2 Ma): grassy woodland – wooded grassland (ca. 20 – 67 % C4 grasses, mean=ca. 42 %)
- Mean pedogenic carbonate $\delta^{13}C$ in Lower Nawata: -5; corresponds to ca. 50% C4 biomass (*97*)

- Nkondo-Kaiso, Uganda (Nkondo Formation) 6-5 Ma
  - "Higher rain than today and much more diverse; different vegetation in close proximity; locally humid" (*130* supplement, *144, 145*)
  - Pickford et al. (*146*):
    - Fossil plants, especially wood are abundant
    - In the Late Miocene the faunal and floral elements indicate dense semi-deciduous forest environment
    - Fauna can be most closely correlated with that of Lukeino; proboscideans represented by *Mammuthus subplanifrons*, *Anancus kenyensis*, *Stegodon kaisensis* and early *Loxodonta* (cf. *L. cookei* (*15*)).
  - Deschamps and Ergo (*145*):
    - Fruit fossils indicated tropical forest (*144*), but fossil wood indicates a more complex mosaic of forest and wooded savanna
  - Boaz (*147*):
    - Faunas emphasize "humid", forested, or well-wooded characteristics (e.g. *148*)
    - Analysis of flora (*144*) "indicates the presence of lowland forest similar to that in modern northern Kasai, Zaire" (=Salonga National Park, Democratic Republic of Congo)
  - Cote (*149*):
    - "Humid environment with mosaic of forest and savannah. Also supported by fossil woods and fruits." (*144*)
    - "Gorilla suggests well-forested environment"
  - Pollen records indicate on average ca. 15 % proportion of grasses ("Graminidites") in the vegetation (*150*)
- Manonga Valley, Ibole Member, Tanzania (5.5 – 5 Ma)
  - Cote (*149*):

        - "Mosaic of more open and more bushy habitats near lake. Lack of arboreal taxa suggests no closed forest"
        - However: taphonomical bias towards large-sized mammals (could this explain the lack of arboreal taxa?)
    - Harrison and Baker (*151*):
        - The fauna is dominated by proboscideans and suids, which indicate that well-wooded habitats dominated near the Manonga lake
    - Overall interpretation: woodland or shrubland with more open areas nearby, dense forest not likely.

PLIOCENE

- Kanapoi (4.2 – 4.1 Ma) (*152*)
    - Predominantly semi-arid grassy woodland/shrubland savannah based on stable isotopes from soil carbonates; the dominating Abilat and Dite paleosols indicate ca. 30 – 45 % C4 grasses
    - However, there were also C3 dominated edaphic grasslands with mixed C3 and C4 ephemeral forbs and grasses as indicated by Aberegaiya paleosols (20 – 30 % C4) and some gallery woodland as indicated by Akai-Ititi paleosols (ca. 25 % C4)
    - Herbaceous vegetation grass-dominated, among many forbs, with scattered *Acacia* shrubs and low trees (*Acacia – Commiphora* wooded steppe) might be the best analogue
    - "the most ancient known occurrence of early hominids (*Australopithecus anamensis*) in dry, open habitat"
- Laetoli, Upper Laetoli Beds (3.75 – 3.5 Ma)
    - Woodland and riparian forests (*153*), some grassy wetland areas (*154*)
    - Mixed altitude: forest, dry open woodland, wetland *(101, 130* supplement, *155*)
    - Rossouw and Scott (*156*):
        - Phytoliths indicate "presence of heterogenous habitats throughout the sequence"; "upper part of the phytolith sequence indicates a marked change in the grass component toward C4 dominance and more grassy vegetation"
        - Not enough pollen for reliable vegetation proxy
    - Bamford (*101*):
        - Fossil flora falls into period of 3.8 – 3.5 Ma (Upper Laetoli)
        - "a variety of mesic and dry woodland plants and some forest elements"
        - "grasslands were ubiquitous, but never dominant part of the vegetation"

- Bamford (*155*):
  - Fossil wood analysis indicates “a variety of soils and vegetation associations from forest, evergreen woodland, dry woodland, bushland, riverine and wooded grassland, in a megathermal environment”
  - “Well wooded environment”
- Reed and Denys (*157*):
  - Micromammal fauna supports the interpretation of woodland environment for Upper Laetoli Beds
  - Ndolanya Beds: more mesic environment than Upper Laetoli Beds (?), but smaller proportion of arboreal rodents than in Upper Laetoli Beds
- Large mammals and environment:
  - Stable isotopes of all large herbivores indicate “mixed grazing/browsing strategies or exclusive reliance on C3 browse, more consistent with wooded than grassland-savanna biomes” (*158*)
  - Mesowear indicates that “the almost complete absence of grazing guilds, and the heavy reliance on browse by most fossil herbivores, do not support the inference that the Laetoli environment was dominated by grassland” … “*Eurygnathohippus* sp. is the only dedicated grazer in Laetoli” (*159*)
  - Fossil antelope habitat preferences (based on modern analogues) indicate the continuous regional presence of woodland and forest throughout Upper Laetoli Beds (*160*)
  - Kovarovic and Andrews (161):
    - Postcranial analysis of bovids “indicate that Upper Ndolanya Beds represent a more open environment than was present during the deposition of Upper Laetoli Beds one million years earlier”
    - Upper Laetoli Beds: predominantly heavy woodland-bushland indicated (with various environmental elements, including forest)
    - Upper Ndolanya Beds: predominantly wooded-bushed grassland indicated (with abundant light woodland-bushland elements)
- Plummer et al. (*131*) (based on $\delta^{13}C$ of soil carbonates):
  - Lower Laetoli Beds: grassy woodland (ca. 27 – 50 % C4 grasses)
  - Upper Laetoli Beds: grassy woodland (ca. 30 – 55 % C4 grasses)
  - Ndolanya Beds: grassy woodland – open grassland, mostly wooded grassland (ca. 40 – 87 % C4 grasses)
  - Ngoloba Beds: open grassland (ca. 80 – 85 % C4 grasses)

- Chemeron Formation (Tugen Hills), 5 – 2.4 Ma
  - Kingston et al. (*90*) (based on $\delta^{13}C$ of soil carbonates):

        - Heterogenous vegetation with a mixture of C3 and C4 plants
        - Lower Chemeron (5 – 4 Ma): grassy woodland (ca. 22 – 45% C4 grasses)
        - Middle Chemeron (4 – 3 Ma): forest/woodland – wooded grassland, mostly grassy woodland (ca. 18 – 55 % C4)
        - Upper Chemeron (3 – 2 Ma): woodland – wooded grassland, mostly grassy woodland (ca. 20 – 68 % C4)
- Nkondo-Kaiso, Uganda, Warwire Formation (3.5 – 3 Ma)
    - Pollen records indicate the presence of on average ca. 17 % grasses (“Graminidites”) in vegetation (*150*)
- East Turkana
    - Markedly dryer conditions than in surrounding areas between 4 – 2 Ma, especially during the Moiti Floodplain (4 – 3.6 Ma) and Tulu Bor (3.4 -2 Ma) phases, but not before or since (*42*)
    - Plummer et al. (*131*) based on $\delta^{13}C$ of soil carbonates:
        - Moiti to Burgi: forest/woodland – grassy woodland (ca. 17 – 45 % C4 grasses in biomass)
    - Levin et al. (*162*) based on soil carbonates:
        - Moiti (3.96 Ma): forest/woodland – grassy woodland (ca. 10 – 35 % C4 grasses, mean=ca. 20 %)
        - Lokochot (3.52 – 3.44 Ma): grassy woodland (ca. 25 – 52 % C4 grasses, mean=ca. 35 %)
        - Tulu Bor (3.7 Ma): grassy woodland – wooded grassland (ca. 20 – 58 % C4 grasses, mean=ca. 40 %)
        - Tulu Bor (3.42 – 3.22 Ma): grassy woodland (ca. 22 – 45 % C4 grasses, mean=ca. 32 %)
- West Turkana
    - Levin et al. (*162*) supplement:
        - Lower Lomekwi (3.44 – 3 Ma): soil carbonates: -6 = ca. 45% C4 plants
        - Middle Lomekwi (3 – 2.6 Ma): soil carbonates: -5.1 = ca. 50% C4 plants
        - Upper Lomekwi (2.6 – 2.47 Ma): soil carbonates: -6.5 = ca. 40 % C4 plants
        - Lokalalei (2.4 – 2.34 Ma): soil carbonates: -6.6 = ca. 38 % C4 plants
    - Du et al. (*163*):
        - Lower and Middle Lomekwi: soil carbonates: -7.07 = 35 % C4 plants (grassy woodland)
        - Upper Lomekwi: soil carbonates: -6.64 = ca. 40 % C4 plants (grassy woodland)

    - Lomekwi 3 (ca. 3.3 Ma)
        - stable carbon isotopes indicate a heavily wooded grassland environment; $\delta^{13}C$ = -7.3 ± 1.1 ‰, corresponding with.47 ± 9 % woody cover (*164*)
        - woodland/bushland/thicket/shrubland environment (*164*)
    - All Lomekwi sequence (ca. 3.3 – 2.5 Ma)
        - Mostly woodlands and gallery forests, but also open arid habitats, based on bovid faunas and soil carbon isotopes (*164-166*)
        - Lomekwi 5: based on fauna and geology, “a mosaic of habitats with woodland and forest-edge habitats dominating” (*167*)

PLEISTOCENE

- Koobi Fora
    - Upper Burgi 1.95 Ma (Area 41)
        - Riverine forest, wetter than today (*130, 168*); “predominantly riverine woodland, woodlands and wooded grasslands” …but probably grasslands in the vicinity based on the presence of *Acacia sieberiana*, mammal fauna and soil isotopes (*168*)
    - Plummer et al. (*131*) based on $\delta^{13}C$ of soil carbonates:
        - Lower KBS: forest/woodland – grassy woodland (ca. 20 – 45 % C4 grasses in biomass)
        - Upper KBS: grassy woodland – open grassland, mostly wooded grassland (ca. 40 – 90 % C4 grasses in biomass)
        - Chari, Okote: grassy woodland – open grassland, mostly wooded grassland (ca. 40 – 85 % C4 grasses in biomass)
    - Levin et al. (*162*) based on soil carbonates:
        - Upper Burgi (1.91 – 1.87 Ma): grassy woodland – wooded grassland (ca. 25 – 62 % C4 grasses, mean=ca. 47 %)
        - KBS (1.85 – 1.7 Ma): grassy woodland – wooded grassland (ca. 30 – 70 % C4 grasses, mean=ca. 46 %)
        - Okote (1.46 Ma): grassy woodland – open grassland (ca. 28 – 80 % C4 grasses, mean=ca. 55%)
        - Chari (0.8 – 0.6 Ma): grassy woodland – wooded grassland (ca. 46 – 70 % C4 grasses, mean=ca. 60%)
    - Isaac and Isaac (*169*):
        - Pollen data (*170*) indicates the following overall conclusions of palaeovegetation:
            - Throughout the Koobi Fora Formation, “hills and highlands beyond the basin margins supported communities that contained larger stands of montane forest than exist today”

        - Several "steppe" communities on the floor of the basin: reeds, cattails and grassland along the lake margin; open savannah grassland, wooded grassland and bushland on the floodplain; with gallery woodlands fringing channel courses
    - Lower Burgi Member
        - Areas 102 and 110: pollen indicates "fairly humid conditions at the basin margins, with grasslands along the lake, and open *Acacia-Commiphora* bushland on the plains" (*170*)
    - Upper Burgi Member
        - Pollen is "dominated by grass and herbs, suggesting open, probably arid conditions near the lake, with few indications of highland forest vegetation" (*170*)
    - KBS Member
        - Pollen indicates "cold and semi-humid conditions in the highlands, with lake margin vegetation from salt-loving communities to grassland, while the plains supported an open *Acacia-Commiphora* bushland, and riparian gallery woodland" (*170*)
    - Okote Member: Koobi Fora (Areas 101-103, 106), Illeret (Areas 1-9) and Karari Escarpment (Areas 129-131, 118)
        - Pollen indicates "cold (?) and semi-humid highland vegetation similar to Zone C, with more open, grassy vegetation, including *Commiphora* thorn scrub on the alluvial floodplains, with cattails and sedges along the lake margins and swamps" (*170*)
        - Lower Okote Member:
            - Illeret: "more densely vegetated, including patches of woodland and forest"
            - Karari: "drier bush habitats and riparian woodlands"
            - Koobi Fora: "open lake-margin grassland"

- West Turkana
    - Nariokotome Member (1.3 – 0.7 Ma)
        - Soil carbonates: -4.0 = ca. 60% C4 plants (wooded grassland) (*162*)
        - Soil carbonates: -4.5 = ca. 55% C4 plants (wooded grassland) (*163*)
- Olduvai
    - Plummer et al. (*131*) (based on $\delta^{13}C$ of soil carbonates):
        - Bed I: grassy woodland – wooded grassland (ca. 40 – 60 % C4 grass biomass)
        - Bed II: grassy woodland – open grassland, mostly wooded grassland (ca. 50 – 65 % (35 – 80 % in Lemuta) grass biomass)

        - Bed III: wooded – open grassland (ca. 60 – 87 % C4 grass biomass, similar to the average of Kanjera)
        - Bed IV: wooded grassland (ca. ca. 50 – 65 % C4 grass biomass)
    - 33% - 55% (44 % mean) of grasses and grasses/sedges in the phytolith record of HWK E (Olduvai uppermost bed 1 or lower bed II?) (*171*)
    - ca. 20 % grasses in FLK N 1 (Olduvai Bed I) based on pollen record (*117*, *171*)
- Kanjera
    - On average open grassland to wooded grassland environments, with some indication of grassy woodland, based on pedogenic carbonate isotopes, ca. 75 % C4 grasses on average (70 – 100 %, with some ca. 40 %) (*131*)

RECENT

- Tsavo East National Park, Kenya
    - Grassy woodland – wooded grassland
        - A 400-year pollen record from Kanderi swamp shows a locally grass-dominated environment (local edaphic grassland) (*172*)
        - Mostly semi-arid nyika vegetation, dominated by *Acacia-Commiphora* woodland, including open *Acacia* savanna (*173*)
- Gola Forest, Sierra Leone
    - Tropical lowland rainforest (*174, 175*)
- Ituri-Bwamba Forest, Democratic Republic of Congo and Uganda
    - Tropical forests, largely lowland rainforest
    - Pollen records from Congolean rainforests indicate a presence of 5 % grasses in vegetation (*176*).
- Lake Mai-Ndombe, Democratic Republic of Congo
    - Tropical swamp forests and lowland rainforests (*177*)
    - Pollen records from Congolean rainforests indicate a presence of 5 % grasses in vegetation (*176*).
- Yassonkon near the River Nyong, Cameroon
    - Tropical lowland rainforest (Zoological Museum of Copenhagen, specimen information for *Loxodonta cyclotis* C.N.2980, 2981)

**References (81 – 177)**


81. Tassy, P. Who is who among the Proboscidea? In *The Proboscidea: Evolution and Palaeoecology of Elephants and Their Relatives*, (eds. Shoshani, J., Tassy, P.) 39–48 (Oxford University Press, 1996).
82. Shoshani, J., Ferretti, M.P., Lister, A.M., Agenbroad, L.D., Saegusa, H., Mol, D., & Takahashi, K. Relationships within the Elephantinae using hyoid characters. *Quaternary International* **169-170**, 174–185 (2007).
83. Tassy, P. Les proboscidiens (Mammalia) fossiles du rift occidental, Ouganda. In *Geology and Paleobiology of the Albertine Rift valley, Uganda-Zaire. Vol.II: Paleobiology. Centre International pour la Formation et les Echanges géologiques* (eds. Senut B., Pickford M.). *Publication occasionnelle, Orléans* **29**, 217–257 (1994).
84. Tassy, P. Miocene Elephantids (Mammalia) from the Emirate of Abu Dhabi, United Arab Emirates: Palaeobiogeographic Implications. In *Fossil Vertebrates of Arabia.* (eds. Whybrow P. J., Hill A.) 209–233 (Yale University Press, New Haven, 1999).
85. Saegusa, H., Nakaya, H., Kunimatsu, Y., Nakatsukasa, M., Tsujikawa, H., Sawada, Y., Saneyoshi, M., & Sakai, T. Earliest elephantid remains from the late Miocene locality, Nakali, Kenya. Scientific Annals, School of Geology Aristotle University of Thessaloniki, Greece. *VIth International Conference on Mammoths and Their Relatives, Grevena-Siatista, Special Volume 102,* **175** (2014).
86. Zhang, H. Evolution and systematics of the Elephantidae (Mammalia, Proboscidea) from the Late Miocene to Recent. PhD thesis, University of Bristol, 380 p. (2020).
87. N. Inuzuka, & Takashi, K. Discrimination between the genera *Palaeoloxodon* and *Elephas* and the independent taxonomical position of *Palaeoloxodon* (Mammalia: Proboscidea). *Miscelánea en homenahe a Emiliano Aguirre,* **4-2**, 234–245 (2004).
88. Meyer, M., Palkopoulou, E., Baleka, S., Stiller, M., Penkman, K.E.H., Palaeogenomes of Eurasian straight-tusked elephants challenge the current view of elephant evolution. *eLife 2017* **6**, e25413 (2017).
89. Palkopoulou, E. *et al*. A comprehensive genomic history of extinct and living elephants. *Proc. Natl. Acad. Sci. USA* **115**, E2566-E2574 (2018).
90. Kingston, J.D., Marino, B.D., & Hill, A. Isotopic evidence for Neogene hominid paleoenvironments in the Kenya Rift Valley. *Science* **264**, 955–959 (1994).
91. Roche, D., Ségalen, L., Senut, B., & Pickford, M. Stable isotope analyses of tooth enamel carbonate of large herbivores from the Tugen Hills deposits: Palaeoenvironmental context of the earliest Kenyan hominids. *Earth and Planetary Science Letters* **381**, 39–51 (2013).
92. Andrews, P., & Van Couvering, J.A.H. Palaeoenvironments in the East African Miocene. In *Approaches to primate paleobiology* (ed. F.S. Szalay), 62–103, (Basel, Karger, 1975).
93. Collinson, M.E., Andrews, P., & Bamford, M.K. Taphonomy of the early Miocene flora, Hiwegi Formation, Rusinga Island, Kenya. *Journal of Human Evolution* **57**, 149–162 (2009).
94. Jacobs, B.F. Palaeobotanical studies from tropical Africa: relevance to the evolution of forest, woodland and savannah biomes. *Philosophical Transactions of the Royal Society of London B* **359**, 1573–1583 (2004).

95. Maxbauer, D.P., Peppe, D.J., Bamford, M., McNulty, K.P., Harcourt-Smith, W.E.H., & Davis, L.E. A morphotype catalog and paleoenvironmental interpretations of early Miocene fossil leaves from the Hiwegi Formation, Rusinga Island, Lake Victoria, Kenya, *Palaeontologia Electronica* **16**, Issue 3; 28A; 19p, palaeo-electronica.org/content/2013/547-rusinga-island-flora (2013).
96. Grossman, A., Liutkus-Pierce, C., Kyongo, B., & M'Kirera, F. 2014. New fauna from Loperot contributes to the understanding of Early Miocene catarrhine communities. *International Journal of Primatology* **35**, 1253–1274 (2014).
97. Uno, K.T., Polissar, P.J., Jackson, K.E., & deMenocal, P.B. Neogene biomarker record of vegetation change in eastern Africa. *Proc. Natl. Acad. Sci. USA* **113**, 6355–6363 (2016).
98. Tassy, P., & Pickford, M. Un nouveau mastodonte zygolophodonte (Proboscidea, Mammalia) dans le Miocène inférieur d'Afrique orientale: Systématique et paléoenvironnement. *Geobios* **16**, 53-77 (1983).
99. Chaney, R.W. A Tertiary flora from Uganda. *Journal of Geology* **41**, 702–709 (1933).
100. Hamilton, A.C. Some plant fossils from Bukwa. *Uganda Journal* **32**, 157–164 (1968).
101. Bamford, M. Fossil leaves, fruits and seeds. In *Paleontology and Geology of Laetoli: Human Evolution in Context. Volume 1: Geology, Geochronology, Paleoecology and Paleoenvironment, Vertebrate Paleobiology and Paleoanthropology* (ed. Harrison, T.) 235–252, DOI 10.1007/978-90-481-9956-3_11 (Springer, 2011).
102. Pickford, M. Early Miocene grassland ecosystem at Bukwa, Mount Elgon, Uganda. *Comptes Rendus Palevol* **1**, 213–219 (2002).
103. Kunimatsu, Y., Tsujikawa, H., Nakatsukasa, M., Shimizu, D., Ogihara, N., Kikuchi, Y., Nakano, Y., Takano, T., Morimoto, N., & Ishida, H. A new species of *Mioeuoticus* (Lorisiformes, Primates) from the early Middle Miocene of Kenya. *Anthropological Science* **125**, 59–65 (2017).
104. Suzuki, M. A preliminary description of fossil woods collected from Site BG-X, west of Baragoi, Kenya. *African Study Monographs, Supplementary Issue* **5**, 163–167 (1987).
105. Tsujikawa H., & Nakaya H. Geologic age and palaeoenvironments of mammalian faunas from Samburu Hills, Northern Kenya. *Chikyu Monthly* **27**, 603–611 (2005).
106. Mihlbachler, M.C., McCrossin, M.L., & Benefit, B.R. Body size distribution and habitat structure of Maboko Island, Middle Miocene, Kenya. *Journal of Vertebrate Paleontology* **18**, *Supplement. Abstracts of Papers. Fifty-Eighth Annual Meeting, Society of Vertebrate Paleontology* (Sep. 15, 1998) A1–A94 (1998).
107. Koch, P. Isotopic reconstruction of past continental environments. *Annual Review of Earth and Planetary Sciences* **26**, 573–613 (1998).
108. MacFadden, B.J. Origin and evolution of the grazing guild in Cenozoic New World terrestrial mammals. In *Evolution of Herbivory in Terrestrial Vertebrates: Perspectives from the Fossil Record* (ed. Sues, H.D.) 223–243 (Cambridge, Cambridge University Press, 2000).
109. Retallack, G.J., Wynn, J.G., Benefit, B.R., & McCrossin, M.L. Paleosols and paleoenvironments of the middle Miocene, Maboko Formation, Kenya. *Journal of Human Evolution* **42**, 659–703 (2002).
110. Pickford, M. Preliminary Miocene mammalian biostratigraphy for western Kenya. *Journal of Human Evolution* **10**, 73–97 (1981).

111. Wynn, J.G. & Retallack, G.J. Paleoenvironmental reconstruction of middle Miocene paleosols bearing *Kenyapithecus* and *Victoriapithecus*, Nyakach Formation, southwestern Kenya. *Journal of Human Evolution* **40**, 263–288 (2001).
112. Arney, I., Benefit, B., McCrossin, M.L., MacLatchy, L., Kingston, J.D. Herbivore isotopic dietary ecology of the middle Miocene Maboko Formation, Kenya. *Palaeogeography, Palaeoclimatology, Palaeoecology* **601**, 111061 (2022).
113. Pickford, M. Suidae and Hippopotamidae from the Middle Miocene of Kipsaraman, Kenya and other sites in East Africa. *Paleontological Research* **11**, 85–105 (2007).
114. Pickford, M. Geology and fauna of the middle Miocene hominoid site at Muruyur, Baringo District, Kenya. *Human Evolution* **3**, 381–390 (1988).
115. Behrensmeyer, A.K., Deino, A.L., Hill, A., Kingston, J.D., & Saunders, J.J. Geology and geochronology of the Middle Miocene Kipsaramon site complex, Muruyur Beds, Tugen Hills, Kenya. *Journal of Human Evolution* **42**, 11–38 (2002).
116. Shipman, P. Paleoecology at Fort Ternan reconsidered. *Journal of Human Evolution* **15**, 193–204 (1986).
117. Bonnefille, R. Cenozoic vegetation and environments of early hominoids in East Africa. In *The Evolution of the East Asian Environment, Vol. 2. Palaeobotany, Palaeozoology, and Palaeoanthropology* (ed. Whyte, R.O.) 579–612 (Hong Kong: Center for Asian Studies, University of Hong Kong, 1984).
118. Cerling, T.E., Quade, J., Ambrose, S.H. & Sikes, N.E. Fossil soils from Fort Ternan, Kenya: grassland or woodland? *Journal of Human Evolution* **21**, 295–306 (1991).
119. Retallack, G.J. Middle Miocene fossil plants from Fort Ternan (Kenya) and evolution of African grasslands. *Paleobiology* **18**, 383–400 (1992).
120. Dugas, D.P. & Retallack, G.J. Middle Miocene fossil grasses from Fort Ternan, Kenya. *Journal of Paleontology* **67**, 113–128 (1993).
121. Jacobs, B.F., & Kabuye, C.H.S. A middle Miocene (12.2 my old) forest in the East African Rift Valley, Kenya. *Journal of Human Evolution* **16**, 147-155 (1987).
122. Jacobs, B.F., & Winkler, D.A. Taphonomy of a middle Miocene autochthonous forest assemblage, Ngorora Formation, central Kenya. *Palaeogeography, Palaeoclimatology, Palaeoecology* **99**, 31–40 (1992).
123. Rasmussen, C., Reichenbacher, B., Lenz, O., Altner, M., Penk, S.B.R., Prieto, J., Brüsch, D. Middle–late Miocene palaeoenvironments, palynological data and a fossil fish Lagerstätte from the Central Kenya rift (East Africa). *Geological Magazine* **154**, 24–56 (2017).
124. Sakai, T., Saneyoshi, M., Tanaka, S., Sawada, Y., Nakatsukasa, M., Mbua, E., & Ishida, H. Climate shift recorded at around 10 Ma in Miocene succession of Samburu Hills, northern Kenya Rift, and its significance. In *Monsoon Evolution and Tectonics—Climate Linkage in Asia* (ed. Clift, P. D., Tada, R. and Zheng, H.) *Geological Society of London, Special Publications* **342**, 109–127 (2010).
125. Tsujikawa, H. The palaeoenvironment of *Samburupithecus kiptalami* based on its associated fauna. *African Study Monographs Supplementary Issue* **32**, 51–62 (2005).
126. Ishida, H. Outline the Third Season, 1984, of the Palaeoanthropological Expedition Team to the Samburu Hills and Nachola Areas, Northern Kenya. *African Study Monographs, Supplementary Issue* **5**, 1–6 (1987).

127. Jacobs, B.F., Pan, A.D., & Scotese, C.R. A Review of the Cenozoic Vegetation History of Africa. In *Cenozoic Mammals of Africa* (eds. Werdelin, L., Sanders, W.J.) 57–76 (University of California Press, 2010).
128. Morgan, M.E., Kingston, J.D., Marino, B.D. Expansion and emergence of C4 plants. *Nature* **367**, 162–165 (1994).
129. Kingston, J.D., Jacobs, B.F., Hill, A., Deino, A. Stratigraphy, age and environments of the late Miocene Mpesida Beds, Tugen Hills, Kenya. *Journal of Human Evolution* **42**, 95–116 (2002).
130. Bamford, M., Senut, B., & Pickford, M. Fossil leaves from Lukeino, a 6-million-year-old Formation in the Baringo Basin, Kenya. *Geobios* **46**, 253-272 (2013).
131. Plummer, T., Bishop, L.C., Ditchfield, P., & Hicks, J. Research on Late Pliocene Oldowan sites at Kanjera South, Kenya. *Journal of Human Evolution* **36**, 151–170 (1999).
132. Ambrose, S.H., Nyamai, C.M., Mathu, E.M., & Williams, M.A.J. Geology, geochemistry, and stratigraphy of the Lemudong'o Formation, Kenya Rift Valley. *Kirtlandia* **56**, 53–64 (2007).
133. Hlusko, L.J. Earliest evidence for *Atherurus* and *Xenohystrix* (Hystricidae, Rodentia) in Africa, from the late Miocene site of Lemudong'o, Kenya. *Kirtlandia* **56**, 86–91 (2007).
134. Harris, J.M. Family Suidae. In *Koobi Fora Research Project: Volume II: The Fossil Ungulates, Proboscidea, Perissodactyla, and Suidae* (ed. Harris, J.M.) 215–300 (Oxford University Press, Oxford, 1983).
135. Pickford, M. Fossil Suidae of the Albertine Rift, Uganda-Zaire. In *Geology and Palaeobiology of the Albertine Rift Valley, Uganda-Zaire, Vol. II: Palaeobiology* (ed. Senut, B., & Pickford, M.) 339–373 (*Occasional Publication/International Center for Training and Exchanges in the Geosciences*, **29**. Orleáns, France, 1994).
136. Harris, J.M., & Cerling, T.E. Dietary adaptations of extant and Neogene African suids. *Journal of Zoology* **256**, 45–54 (2002).
137. Hlusko, L.J., & Haile-Selassie, Y. *Nyanzachoerus syrticus* (Artiodactyla, Suidae) from the late Miocene of Lemudong'o, Kenya. *Kirtlandia* **56**, 152–157 (2007).
138. Pickford, M., Hlusko, L.J. Late Miocene procaviid hyracoids (Hyracoidea: *Dendrohyrax*) from Lemudong'o, Kenya. *Kirtlandia* **56**, 106–111 (2007).
139. Manthi, F.K. A preliminary review of the rodent fauna from Lemudong'o, southwestern Kenya, and its implication to the late Miocene paleoenvironments. *Kirtlandia* **56**, 92–105 (2007).
140. Hlusko, L.J. A new late Miocene species of *Paracolobus* and other Cercopithecoidea (Mammalia: Primates) fossils from Lemudong'o, Kenya. *Kirtlandia* **56**, 72–85 (2007).
141. Hlusko, L.J., Haile-Selassie, Y., & Degusta, D. Late Miocene Bovidae (Mammalia: Artiodactyla) from Lemudong'o, Narok District, Kenya. *Kirtlandia* **56**, 163–172 (2007).
142. Bernor, R.L. The latest Miocene hipparionine (Equidae) from Lemudong'o, Kenya. *Kirtlandia* **56**, 148–151 (2007).
143. M. Leakey, J. Harris, *Lothagam - The Dawn of Humanity in Eastern Africa.* 680 p. (Columbia University Press, 2003).
144. Dechamps, R., Senut, B., & Pickford, M. Fruits fossiles pliocènes et pléistocènes du Rift Occidental Ougandais. Signification paléoenvironnementale. *Comptes Rendus de l'Académie des Sciences, Série II* **314**, 325–331 (1992).

145. Deschamps, R., & Ergo, A. Paleovegetation (fossil plants) of the Albertine Rift Valley. In *Geology and palaeobiology of the Albertine Rift Valley, Uganda-Zaire. Volume 2, Palaeobiology/Paléobiologie.* (ed. Pickford, M., & Senut, B.), 29–45 (Orléans, France, 1994).
146. Pickford, M., Senut, B., Hadoto, D. *Geology and palaeobiology of the Albertine Rift valley, Uganda-Zaire. Vol. I: geology. Occasional Publication/International Center for Training and Exchanges in the Geosciences*, **24** (Orleáns, France, 1993).
147. Boaz, N.T. Significance of the Western rift for hominin evolution. In *Integrative Paths to the Past: Paleoanthropological Advances in Honor of F.Clark Howell* (ed. Corruccini, R.L., Ciochon, R.L.) 321–343 (Prentice Hall, Englewoods Cliffs, 1994).
148. Pickford, M. Tempo and mode of molluscan evolution in the Pliocene of the Albertine Rift, Uganda-Zaire. *Comptes rendus de l'Académie des sciences. Série II. Mechanics. Physics. Chemistry. Space sciences. Earth sciences* **311**, 1103–1109 (1990).
149. Cote, S.M. Origins of the African hominoids: an assessment of the palaeobiogeographical evidence. *Comptes Rendus Palevol* **3**, 323–340 (2004).
150. Lukaye, J.M. Biostratigraphy and Palynofacies of Four Exploration Wells from the Albertine Graben, Uganda. *Search and Discovery Article 50169, Adapted from oral presentation at AAPG International Conference and Exhibition* (Cape Town, South Africa, 2009).
151. Harrison, T., Baker, E. Paleontology and biochronology of fossil localities in the Manonga Valley, Tanzania. In *Neogene Paleontology of the Manonga Valley, Tanzania; Volume 14 of Topics in Geobiology* (ed. Harrison, T.) 378–381 (Plenum Press, New York, 1997).
152. Wynn, J.G. Paleosols, stable carbon isotopes, and paleoenvironmental interpretation of Kanapoi, Northern Kenya. *Journal of Human Evolution* **39**, 411–432 (2000).
153. Andrews, P., & Bamford, M. Past and present vegetation ecology of Laetoli, Tanzania. *Journal of Human Evolution* **54**, 78–98 (2008).
154. R. Bonnefille, G. Riollet. Palynological spectra from the Upper Laetolil Beds. In *Laetoli: A Pliocene Site in Northern Tanzania*, (eds. Leakey, M.D., Harris, J.H.) 52–61(Oxford, Clarendon Press, 1987).
155. Bamford, M. Fossil woods. In *Paleontology and Geology of Laetoli: Human Evolution in Context. Volume 1: Geology, Geochronology, Paleoecology and Paleoenvironment, Vertebrate Paleobiology and Paleoanthropology* (ed. Harrison, T.) 217–233 (Springer, 2011).
156. Rossouw, L., Scott, L. Phytoliths and pollen, the microscopic plant remains in Pliocene volcanic sediments around Laetoli, Tanzania. In *Paleontology and Geology of Laetoli: Human Evolution in Context. Volume 1: Geology, Geochronology, Paleoecology and Paleoenvironment, Vertebrate Paleobiology and Paleoanthropology* (ed. Harrison, T.) 201–215 (Springer, 2011).
157. Reed, D., Denys, C. The taphonomy and paleoenvironmental implications of the Laetoli micromammals. In *Paleontology and Geology of Laetoli: Human Evolution in Context. Volume 1: Geology, Geochronology, Paleoecology and Paleoenvironment, Vertebrate Paleobiology and Paleoanthropology* (ed. Harrrison, T.) 265–278 (Springer, 2011).
158. Kingston, J.D. Stable isotopic analyses of Laetoli fossil herbivores. In *Paleontology and Geology of Laetoli: Human Evolution in Context. Volume 1: Geology, Geochronology,*

*Paleoecology and Paleoenvironment, Vertebrate Paleobiology and Paleoanthropology* (ed. Harrison, T.) 293–382 (Springer, 2011).

159. Kaiser, T.M. Feeding ecology and niche partitioning of the Laetoli ungulate faunas. In *Paleontology and Geology of Laetoli: Human Evolution in Context. Volume 1: Geology, Geochronology, Paleoecology and Paleoenvironment, Vertebrate Paleobiology and Paleoanthropology* (ed. Harrison, T.) 329–354 (Springer, 2011).
160. Bishop, L. C., Plummer, T. W., Hertel, F., & Kovarovic, K. Paleoenvironments of Laetoli, Tanzania as Determined by Antelope Habitat Preferences. In *Paleontology and Geology of Laetoli: Human Evolution in Context. Volume 1: Geology, Geochronology, Paleoecology and Paleoenvironment, Vertebrate Paleobiology and Paleoanthropology* (ed. Harrison, T.) 355–366 (Springer, 2011).
161. Kovarovic, K., Andrews, P. Environmental change within the Laetoli fossiliferous sequence: vegetation Catenas and bovid ecomorphology. In *Paleontology and Geology of Laetoli: Human Evolution in Context. Volume 1: Geology, Geochronology, Paleoecology and Paleoenvironment, Vertebrate Paleobiology and Paleoanthropology* (ed. Harrison, T.) 367–380 (Springer, 2011).
162. Levin, N. E., Brown, F. H., Behrensmeyer, A. K., Bobe, R. & Cerling, T. E. Paleosol carbonates from the Omo Group: isotopic records of local and regional environmental change in East Africa. *Palaeogeogr. Palaeoclimatol. Palaeoecol.* **307**, 75–89 (2011).
163. Du, A., Robinson, J.R., Rowan, J., Lazagabaster, I.A. & Behrensmeyer, A.K. Stable carbon isotopes from paleosol carbonate and herbivore enamel document differing paleovegetation signals in the eastern African Plio-Pleistocene. *Review of Palaeobotany and Palynology* **261**, 41-52 (2019).
164. Harmand, S., Lewis, J.E., Feibel, C.S., Lepre, C.J., Prat, S., Lenoble, A., Boes, X., Quinn, R.L., Brenet, M., Arroyo, A., Taylor, N., Clement, S., Daver, G., Brugal, J.-P., Leakey, L., Mortlock, R.A., Wright, J.D., Lokorodi, S., Kirwa, C., Kent, D.V., & Roche, H. 3.3-million-year-old stone tools from Lomekwi 3, West Turkana, Kenya. *Nature* **521**, 310–315 (2015).
165. Harris, J.M., Brown, F.H., Leakey, M.G., Walker, A.C., & Leakey, R.E. Pliocene and Pleistocene Hominid-Bearing Sites from West of Lake Turkana, Kenya. *Science* **239**, 27–33 (1988).
166. Behrensmeyer, A.K., & Reed, K.E. Reconstructing the habitats of *Australopithecus*: paleoenvironments, site taphonomy, and faunas. *In The Paleobiology of Australopithecus. Contributions from the Fourth Stony Brook Human Evolution Symposium and Workshop, Diversity in Australopithecus: Tracking the First Bipeds September 25–28, 2007. (*ed. Reed, K.E., Fleagle, J.G., & Leakey, R.E.) 41–60 (Springer, 2013).
167. Jablonski, N.G., Leakey, M.G., Kiarie, C., & Antón, M. A new skeleton of *Theropithecus brumpti* (Primates: Cercopithecidae) from Lomekwi, West Turkana, Kenya*. Journal of Human Evolution* **43**, 887–923 (2002).
168. Bamford, M.K. Late Pliocene woody vegetation of Area 41, Koobi Fora, East Turkana Basin, Kenya. *Review of Palaeobotany and Palynology* **164**, 191–120 (2011).
169. Isaac, G., & Isaac, B. *Koobi Fora research project, volume 5: Plio-Pleistocene archaeology.* 632 p. (Oxford, UK: Clarendon Press, 1997).
170. Vincens, *A. Palynologie, environements actuels et plio-pleistocene a l'est du lac Turkana (Kenya).* These d'etat-sciences, l'universite d'Aix-Marseille II (1982).

171. Barboni, C., Ashley, G.M., Dominiguez-Rodrigo, M., Brunn, H.T., Mabulla, A.Z.P., & Baquedano, E. Phytoliths infer locally dense and heterogeneous paleovegetation at FLK North and surrounding localities during upper Bed I time, Olduvai Gorge, Tanzania. *Quaternary Research* **74**, 344–354 (2010).
172. Gillson, L. Testing non-equilibrium theories in Savannas: 1400 years of vegetation change in Tsavo National Park, Kenya. *Ecological Complexity* **1**, 281–298 (2004).
173. Leuthold, W., & Sale, J.B. Movements and patterns of habitat utilization of elephants in Tsavo National Park, Kenya. *East African Wildlife Journal* **11**, 369–384 (1973).
174. Cole, N.H.A. The Gola Forest in Sierra Leone: A Remnant of Tropical Primary Forest in need of Conservation. *Environmental Conservation* **7**, 33–40 (1980).
175. Leach, M., & Fairhead, J. Fashioned forest pasts, occluded histories? International environmental analysis in West African locales. *Development and Change* **31**, 35–59 (2000).
176. Brncic, T.M., Willis, K.J., Harris, D.J., & Washington, R. Culture or climate? The relative influences of past processes on the composition of the lowland Congo rainforest. *Phil. Trans. R. Soc. B* **362**, 229–242 (2007).
177. Bwangoy, J.R., Hansen, M., Roy, D., De Grandi, G., Justice, J. Wetland mapping in the Congo Basin using optical and radar remotely sensed data and derived topographical indices. *Remote Sensing of the Environment* **114**, 73–86 (2010).
