# Supplementary material for: Fluctuating climate and dietary innovation drove ratcheted evolution of proboscidean dental traits
Source: Nat Ecol Evol. 2023 Aug 14;7(9):1490–502. doi: 10.1038/s41559-023-02151-4 (PMC10482678; doi:10.1038/s41559-023-02151-4)
Supplement: Supplementary file 2 — Reporting Summary [file 41559_2023_2151_MOESM2_ESM.pdf]

nature portfolio

Corresponding author(s): Juha Saarinen

Last updated by author(s): 19.6.2023

Reporting Summary

Nature Portfolio wishes to improve the reproducibility of the work that we publish. This form provides structure for consistency and transparency in reporting. For further information on Nature Portfolio policies, see our Editorial Policies and the Editorial Policy Checklist.

Statistics

For all statistical analyses, confirm that the following items are present in the figure legend, table legend, main text, or Methods section.

n/a	Confirmed	
☐	☒	The exact sample size (*n*) for each experimental group/condition, given as a discrete number and unit of measurement
☐	☒	A statement on whether measurements were taken from distinct samples or whether the same sample was measured repeatedly
☐	☒	The statistical test(s) used AND whether they are one- or two-sided<br>*Only common tests should be described solely by name; describe more complex techniques in the Methods section.*
☐	☒	A description of all covariates tested
☐	☒	A description of any assumptions or corrections, such as tests of normality and adjustment for multiple comparisons
☐	☒	A full description of the statistical parameters including central tendency (e.g. means) or other basic estimates (e.g. regression coefficient) AND variation (e.g. standard deviation) or associated estimates of uncertainty (e.g. confidence intervals)
☐	☒	For null hypothesis testing, the test statistic (e.g. *F*, *t*, *r*) with confidence intervals, effect sizes, degrees of freedom and *P* value noted<br>*Give P values as exact values whenever suitable.*
☒	☐	For Bayesian analysis, information on the choice of priors and Markov chain Monte Carlo settings
☒	☐	For hierarchical and complex designs, identification of the appropriate level for tests and full reporting of outcomes
☐	☒	Estimates of effect sizes (e.g. Cohen's *d*, Pearson's *r*), indicating how they were calculated

Our web collection on statistics for biologists contains articles on many of the points above.

Software and code

Policy information about availability of computer code

Data collection	NA
Data analysis	R Studio version 3.5.3, 4.2.2, JMP 14, IBM SPSS v. 25, STATISTICA 13.3

For manuscripts utilizing custom algorithms or software that are central to the research but not yet described in published literature, software must be made available to editors and reviewers. We strongly encourage code deposition in a community repository (e.g. GitHub). See the Nature Portfolio guidelines for submitting code & software for further information.

Data

Policy information about availability of data

All manuscripts must include a data availability statement. This statement should provide the following information, where applicable:

- Accession codes, unique identifiers, or web links for publicly available datasets
- A description of any restrictions on data availability
- For clinical datasets or third party data, please ensure that the statement adheres to our policy

All data are provided in Supplementary Data Tables 1-10 and in Figshare online repository (10.6084/m9.figshare.23276126)

Research involving human participants, their data, or biological material

Policy information about studies with human participants or human data. See also policy information about sex, gender (identity/presentation), and sexual orientation and race, ethnicity and racism.

Reporting on sex and gender	NA
Reporting on race, ethnicity, or other socially relevant groupings	NA
Population characteristics	NA
Recruitment	NA
Ethics oversight	NA

Note that full information on the approval of the study protocol must also be provided in the manuscript.

Field-specific reporting

Please select the one below that is the best fit for your research. If you are not sure, read the appropriate sections before making your selection.

☐ Life sciences ☐ Behavioural & social sciences ☒ Ecological, evolutionary & environmental sciences

For a reference copy of the document with all sections, see nature.com/documents/nr-reporting-summary-flat.pdf

Ecological, evolutionary & environmental sciences study design

All studies must disclose on these points even when the disclosure is negative.

Study description	We analyse relationships between proboscidean dental traits, dental mesowear (diet) and environmental proxies (aeolian dust accumulation and locality mean ordinated hypsodonty for aridity, stable carbon isotopes and paleobotanical records for proportion of grass in plant communities), using least squares multiple regressions, multiple regressions commonality analyses, time series breakpoint analyses and partial correlations of dental trait variables. We explore phylogenetic correlations of traits with Pagel's test performed on global proboscidean supretree.
Research sample	The primary research material for this study are morphometric measurements and dental mesowear angles from East African fossil proboscidean molar teeth covering the last 26 million years. For associated data we use various literature sources for estimated percentage of grasses in fossil plant communities (based on paleobotanical and stable isotope records) and aeolian dust accumulation in deep-sea sediments (source literature listed in the supplementary material). In addition, we used NOW-database (https://nowdatabase.org/) for locality mean ordinated hypsodonty values (additional aridity proxy). For Pagel's test of phylogenetic correlation of traits we used proboscidean supertree data published by Cantalapiedra et al. (2021)
Sampling strategy	Availability of fossil material and associated environmental proxy data dictated sample sizes. Sample sizes were not restricted beyond the availability of fossil material and available proxy data.
Data collection	Juha Saarinen (corresponding author) collected the data during research visits to several palaeontological collections. J Saarinen also collected associated paleo-proxy data from literature and database sources.
Timing and spatial scale	The data were collected during museum visits between 2016 and 2019.
Data exclusions	No available data were excluded from the study. In cases where part of the data were excluded from particular analyses, reasons for excluding those data in those particular cases have been justified in the manuscript text.
Reproducibility	All analyses presented in the study can be reproduced using the data are provided in supplementary tables and the analysis methods, software and r-packages described in the manuscript.
Randomization	NA
Blinding	NA

Did the study involve field work? ☐ Yes ☒ No

Reporting for specific materials, systems and methods

We require information from authors about some types of materials, experimental systems and methods used in many studies. Here, indicate whether each material, system or method listed is relevant to your study. If you are not sure if a list item applies to your research, read the appropriate section before selecting a response.

Materials & experimental systems

n/a	Involved in the study
☒	☐ Antibodies
☒	☐ Eukaryotic cell lines
☐	☒ Palaeontology and archaeology
☒	☐ Animals and other organisms
☒	☐ Clinical data
☒	☐ Dual use research of concern
☒	☐ Plants

Methods

n/a	Involved in the study
☒	☐ ChIP-seq
☒	☐ Flow cytometry
☒	☐ MRI-based neuroimaging

Palaeontology and Archaeology

Specimen provenance	The proboscidean fossils used in this study are stored in the following museums and institutes: Natural History Museum, London, UK; Humboldt Museum of Natural History, Berlin, Germany; Central Africa Museum, Tervuren, Belgium; National Museums of Kenya, Nairobi; Kipsaraman Museum, Kipsaraman,Kenya; Tsavo Research Station, Voi, Kenya; Uganda Museum, Kampala; and National Museum of Tanzania, Dar es Salaam). Research permits for studying the materials from Kenya and Tanzania were obtained from NACOSTI and COSTECH, respectively. JJS had research affiliations with the Kenya Wildlife Service, National Museums of Kenya, National Museum of Tanzania and Uganda Museum for the research.
Specimen deposition	All specimens are deposited in the collections of the museums listed above.
Dating methods	This study does not include new dating of the fossil materials. All data on fossil locality ages are based on published literature sources listed in the article and/or supplementary materials.

☒ Tick this box to confirm that the raw and calibrated dates are available in the paper or in Supplementary Information.

Ethics oversight	All museums and institutes storing the fossil materials used in this study agreed to the study protocol. Necessary research permits and affiliations were obtained from the museums and istitutions, and from NACOSTI for research done in Kenya and COSTECH for research done in Tanzania.

Note that full information on the approval of the study protocol must also be provided in the manuscript.
